# Cytokine profile, differential somatic cell count, and oxidative status of Italian Mediterranean buffalo milk affected by the temperature–humidity index

**DOI:** 10.3389/fvets.2024.1449017

**Published:** 2024-11-13

**Authors:** Maria Giovanna Ciliberti, Antonella Santillo, Mariangela Caroprese, Marzia Albenzio

**Affiliations:** Department of Agriculture, Food, Natural Resources, and Engineering (DAFNE), University of Foggia, Foggia, Italy

**Keywords:** immune system, biomarkers, heat stress, somatic cells, animal welfare

## Abstract

In the context of climate change, there has been an increased interest in improving management practices for animals genetically adapted to extreme environmental conditions, such as buffaloes. The temperature–humidity index (THI) is used to determine the severity of heat stress in livestock. This study aimed to evaluate the cytokine profile, oxidative staus, differential somatic cell count (DCC), and the surface expression and activity of myeloperoxidase (MPO) in the somatic cells (SCs) of buffalo. Milk samples (*n* = 216) were collected from the spring to summer season under three different THI classes (THI < 72; ≤72 THI < 76, and THI ≥ 76). The cytokine profile was determined using ELISA, and the expression of DSCC and MPO was determined by flow cytometry. MPO activity was performed on SC extracts using a specific ELISA kit. Oxidative status was determined by the antioxidant/oxidant balance combining the free radical scavenging activity levels, and reactive oxygen and nitrogenous species. The results on the cytokine profile showed that at the THI ≥ 76 the levels of both IL-10 and IFN-γ were highest. IL-1β secretion was lower at the THI < 72 than at the THI values ranging from ≤72 THI < 76. Higher levels of both TNF-α and IL-12 were registered in both THI < 72 and THI ≥ 76 classes. The level of IL-4 was higher in the THI ≥ 76 class than in the ≤72 to <76 range. Data on DCC showed a decrease in the percentage of macrophages and lymphocytes as the THI increased from the ≤72 to <76 range to THI ≥ 76. Furthermore, the highest percentage of polymorphonuclear leukocyte (PMNLs) was registered in both ≤72 to <76 and THI ≥ 76 classes. The MPO activity and surface expression on SC were lower at a THI above 76, which could be associated with an absence of inflammation. A condition of oxidative imbalance was registered as demonstrated by the lower levels of antioxidant/oxidant balance along with increasing THI. Present data demonstrated that buffaloes were able to modulate the alteration of immune response activated by heat stress throughout a series of cross-linked mechanisms involving cytokine networks, different somatic cell distribution, and oxidative status.

## 1 Introduction

Water buffaloes play an important role in the economy of many tropical and subtropical countries contributing to the production of meat and milk with high nutritional value (1). Due to their historical adaptation to hot climates, water buffaloes are better able to tolerate hot and humid conditions than other livestock species (2, 3). Based on these notable characteristics of resilience and adaptability to tropical climates (4), the buffalo represents an important food source of animal origin. However, given the discouraging estimates for future climate scenarios (5, 6), a negative effect of heat stress on buffalo farming is expected and should be properly managed. In particular, it has been demonstrated that exposure to heat stress in buffaloes activates a physiological adaptive response that leads to decreased feed intake, reduced efficiency and utilization of the diet, and alterations in water metabolism, protein, energy, and mineral balance, as well as in enzymatic reactions, hormonal secretions, and blood metabolites (7). This physiological response to thermal adaptation contributes to the reduction in milk yield and quality (8) and the reproductive performance with silent heat and decreased fertility (9). Moreover, exposure to high temperatures impairs lymphocyte function in buffaloes (10), and the reduced cellular immunity is mainly due to an altered T-helper (Th)1/Th2 cytokine balance, favoring the secretion of Th2 cytokines (IL-4, IL-10, and IL-13) (11). A recent comparative study on the survival and phagocytic activity of buffalo and bovine leukocytes following *in vitro* hyperthermia exposure has concluded that buffalo leukocytes exhibit better thermal adaptation than bovine cells, despite a reduction in neutrophil phagocytosis (12). Additionally, during the summer season, buffaloes exhibit oxidative imbalances (13), which also may impair immune function (14). In the previous study by Albenzio et al. (15), different THI classes have influenced buffalo milk production and composition. To the best of our knowledge, there is limited information on the immune response of buffaloes under varying environmental conditions and the complexity of immune mechanisms, particularly the role of cytokines. The hypothesis of this study is that udder health could be affected by adverse temperature and humidity conditions, shifting the cytokine secretion in favor of the Th2 ratio, and causing an oxidative imbalance condition. Therefore, this study aimed to evaluate the cytokine profile, differential somatic cell counts, and the oxidative status of milk across three THI classes observed during the late spring and summer. Additionally, the potential use of myeloperoxidase (MPO) in somatic cells as a biomarker for mammary gland inflammation is discussed.

## 2 Materials and methods

### 2.1 Animals

The details of the experimental trial were previously reported in Albenzio et al. (15). Briefly, 18 Italian Mediterranean buffalo cows balanced for days from parturition (38.5 d) and parity (6.6) were enrolled in the study and raised in a commercial farm located approximately 15 km south-west of Foggia, Apulia, southern Italy (latitude: 41°27′6″N; longitude: 15°33′5″E). The buffaloes were housed in a free stall open-sided barn with a concrete floor, and the resting and feeding areas were covered by a roof. Animals were fed with a concentrated diet mainly composed of corn silage (35.06% on DM), wheat middlings (10.40% on DM), corn flour (10.41% on DM), soy flour (15.61% on DM), vetch and oat hay (15.43% on DM), and straw (13.40% on DM) as extensively reported by Albenzio et al. (15). From the spring (May) to the summer (end of July) 2022 seasons, individual milk samples were collected every 2 weeks for six consecutive samplings, and a total of 216 milk samples were analyzed. Milk samples were defatted by centrifugation at 2,000 *g* for 30 min at 4°C, and the milk whey was collected and stored at −20°C until analysis. Somatic cells (SCs) were then counted and suspended in a freezing medium composed of complete RPMI medium (10% fetal bovine serum-FBS, penicillin–streptavidin, and L-glutamine), along with 10% dimethylsulfoxide for cryopreservation and 50% FBS. The SCs were stored at −80°C until further analyses. Throughout the experiment, environmental temperature and relative humidity (ET and RH) were recorded at a weather station located 10 km from the farm and combined into the temperature humidity index (THI) using the Kelly and Bond (16) formula.

$THI=\left(1.8\times Tdb+32\right)-\left(0.55–0.0055\times RH\right)\times \left(1.8\times Tdb-26\right)$

where Tdb is the dry bulb temperature and RH is the relative humidity.

In order to analyze the effect of the THI on the immunological parameters of buffalo milk, three classes of THI were assigned *a posteriori* to each milk sampling (THI < 72, 72 ≤ THI < 76, and THI ≥ 76), and all data were analyzed according to the rationale reported in Albenzio et al. (15). Temperature (°C, min and max), relative humidity (%, min and max), and the THI (min and max) recorded throughout the experimental trial are reported in Supplementary Table 1. The Institutional Animal Care and Use Committee of the University of Foggia approved all procedures involved on animals with protocol number 0039184-2/2022; furthermore, throughout the experiment the health status of animals was constantly checked by the veterinarian and no signs of any diseases were detected.

### 2.2 Determination of cytokine profile in milk whey

All antibodies and recombinant proteins used were verified for cross-reactivity with buffalo species, with cross-reactivity rates exceeding 90%. All samples were run in duplicate. The evaluation of the concentration of IL-1β and IL-10 cytokines in buffalo milk whey was carried out according to Ciliberti et al. (17) using a sandwich ELISA. Briefly, mouse monoclonal antibodies (mAbs) for ovine IL-1β (Clone 1D4, Bio-Rad Ltd., at a final concentration of 2 μg/mL), and bovine IL-10 (Clone CC318, Bio-Rad Ltd., at a final concentration of 2 μg/mL) were used as capture antibodies. The detecting antibodies were represented by the rabbit polyclonal anti-ovine IL-1β Ab (Polyclonal IgG, Bio-Rad Ltd., at a final concentration of 2 μg/mL) and the biotinylated secondary anti-bovine IL-10 mAb (Clone CC320, Bio-Rad Ltd., at a final concentration of 2 μg/mL). The samples were compared to a standard curve generated using a 4-parameter logistic fit method based on serial dilutions of recombinant bovine IL-1β protein (Kingfisher Biotech Inc., St. Paul, MN, USA, range 500–0 ng/mL, and *R*^2^ = 0.995), which has 99% homology with buffalo IL-1β protein, as stated by the manufacturer. Similarly, recombinant bovine IL-10 (Bio-Rad Ltd.; range: 500–0 ng/mL, *R*^2^ = 0.999) was used, exhibiting 90% homology with buffalo IL-10 protein (UniProt Accession no. P43480). Data were expressed in nanograms of IL-1β and IL-10 per milliliter.

The IFN-γ and TNF-α determination in milk whey was carried out following the Ciliberti et al. (18) ELISA procedure. In brief, the anti-bovine IFN-γ (Clone CC330, Bio-Rad Ltd., at a final concentration of 2 μg/mL) and anti-bovine TNF-α (Kingfisher Biotech, at a final concentration of 5 μg/mL) antibodies were dissolved in coating buffer for the overnight step at 4°C. Biotinylated anti-bovine IFN-γ antibody (Clone CC302, Bio-Rad Ltd., at a final concentration of 2 μg/mL) and biotinylated anti-bovine TNF-α antibody (Kingfisher Biotech, at a final concentration of 2.5 μg/mL) were used as detection antibodies of the sandwich. For the secondary antibody, streptavidin–horseradish peroxidase (HRP) conjugate (Bio-Rad Ltd., diluted 1/500 in PBS) was used for both the IFN-γ and TNF-α assays. A scalar dilution of recombinant bovine TNF-α protein (Kingfisher Biotech, range 2,500–0 ng/mL, *R*^2^ = 0.098), which has 100% homology with buffalo TNF-α protein as per the manufacturer’s instructions, and recombinant bovine IFN-γ protein (Bio-Rad Ltd., range: 5,000–0 pg/mL, *R*^2^ = 0.997) exhibiting 100% homology with buffalo IFN-γ protein (UniProt Accession no. P07353) was added to each plate to create a standard curve (using the four-parameter logistical fit method). Data were expressed as nanograms per milliliter for TNF-α, and the level of IFN-γ was measured as picograms per milliliter.

The levels of IL-4 and IL-12 subunit 40 were performed following the Ciliberti et al. (53) procedure. The antibodies used for sandwich ELISA building were characterized by the mouse anti-bovine IL-4 antibody (Clone CC303, Bio-Rad Ltd., at a final concentration of 2 μg/mL), the mouse anti-bovine IL-4 antibody: biotin (Clone CC314, Bio-Rad Ltd., at a final concentration of 2 μg/mL), the mouse anti-bovine IL-12 low endotoxin (Clone CC301, Bio-Rad Ltd., at final concentration of 2 μg/mL), and the mouse anti-bovine: biotin IL-12 (Clone CC326, Bio-Rad Ltd., at final concentration of 2 μg/mL), respectively. The standard curves were obtained using scalar dilutions of IL-4 bovine recombinant protein (Bio-Rad, range of 500–0 ng/mL and *R*^2^ = 0.994), which has 100% homology with buffalo IL-4 protein (UniProt Accession no. P30367), and IL-12 bovine recombinant protein (Kingfisher Biotech, range 2,500–0 ng/mL and *R*^2^ = 0.993), exhibiting 98% homology with buffalo IL-12 protein, as per the manufacturer’s instructions. All the reads were stopped after 10 min from the addition of the 3,3′,5,5′-tetramethylbenzidine (TMB, Sigma-Aldrich, Milan, Italy) substrate solution and read at 450 nm using a spectrophotometer (Power Wave XS, BioTek). Data were obtained using the 4-parameter logistical fit method and expressed as nanograms per milliliter for both IL-4 and IL-12 cytokines. For all cytokines measured, the inter- and intra-assay coefficients of variability (CV) were less than 15 and 10%, respectively.

### 2.3 Flow cytometric analysis of differential somatic cell count

**Figure 1:**
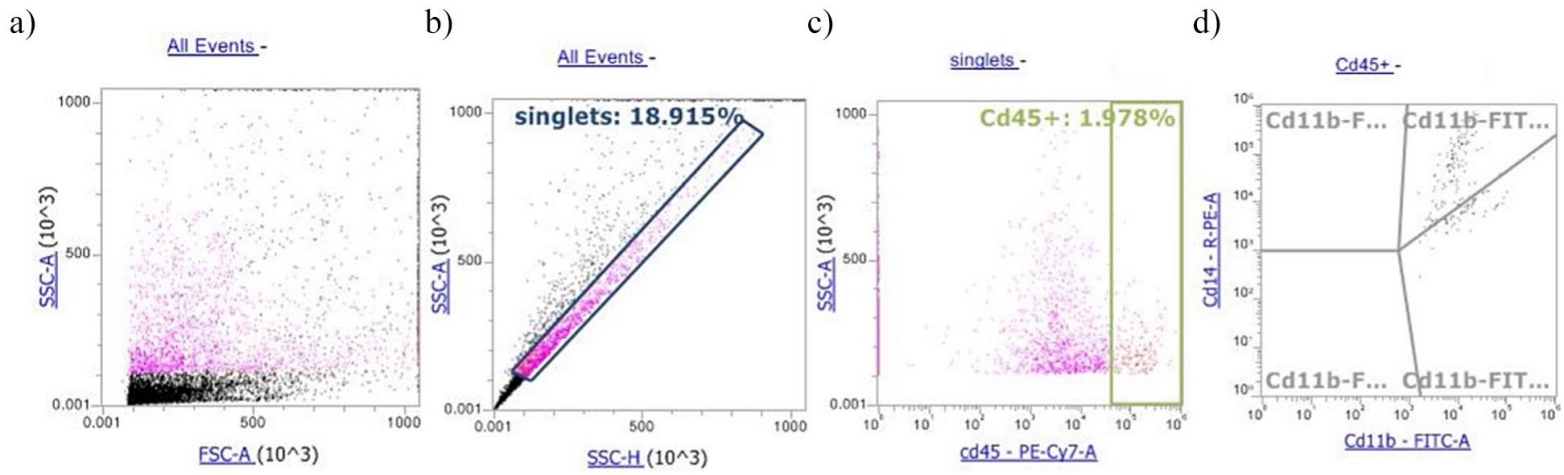
Example of gating strategy for milk buffaloes differential somatic cell count: (a) morphological dot plot, (b) singlets gate, (c) Cd45^+^ somatic cells, and (d) dot plot quadrants: Cd11b^+^/Cd14^+^ cell subset identified macrophages; Cd11b^+^ identifies PMNLs.

Somatic cells were rapidly thawed in a water bath at 37°C and centrifuged at 400×*g* for 15 min. Then, the freezing medium was discarded, and the SCs were suspended in 1% phosphate buffer saline (PBS). Conjugated monoclonal antibodies against CD45-PECy7 (10 μL, Thermo Fisher), and CD11b-FITC (10 μL, Bio-Rad), CD14-PE (5 μL, Bio-Rad) cell subsets, were employed for the identification of milk SCs and added to 100 μL of SC suspension. Tubes were incubated with antibodies for 30 min at room temperature in the dark. Then, after a washing step (1×), SCs were suspended in 500 μL of PBS. Flow cytometry detection was performed by using an Attune NxT Flow Cytometer equipped with a blue laser (Thermo Fisher) according to Schwarz et al. (19). Briefly, 10,000 cells from each sample were differentiated into lymphocytes, olymorphonuclear leukocyte (PMNLs), and macrophages. The PMNLs were measured as CD11b^+^ cells, whereas macrophages were identified as a double-positive subset of CD11b^+^/CD14^+^ cells. Lymphocytes were calculated as the percentage of total leukocytes (CD45^+^) subtracted from PMNLs (% of CD11b^+^) and macrophages (% of CD11b^+^/CD14^+^). Cells with no antibody labeling were used as negative controls to measure the background fluorescence. In addition, due to multiparameter staining, fluorescence minus one (FMO) controls were employed, which included all antibody conjugates except for the specific one being controlled (20). The gating strategy, illustrated in Figure 1, involved the following steps: (1) identifying singlets, (2) defining the dot plot of the population of CD45^+^ cells, which discriminates all the leukocytes present in SCs; and (3) analyzing the CD45^+^ cells to identify the single CD11b^+^ cell subset (PMNLs) and the double-positive CD11b+/CD14+ cells (macrophages). Before immunophenotypization, 7-amino actinomycin D (AAD) DNA strain was added to the cells in a single tube for each sample to assess the viability of SCs, which was found to be greater than 99%. Apart from the immunophenotypization, in order to measure the surface MPO expression, a tube containing a suspension of SCs stained with 5 μL of anti-human MPO-FITC was added and incubated for 30 min at room temperature in the dark. Then, after a washing step (1×), the cells were suspended in 500 μL of PBS. Supplementary Figure 1 shows the morphological dot plot of a positive somatic cell stained with the MPO-FITC antibody.

### 2.4 Determination of myeloperoxidase (MPO) activity in somatic cell extracts

MPO activity was assessed in SC extracts (*n* = 216) following the manufacturer’s suggestion reported in the OxiSelect™ Myeloperoxidase Chlorination Activity Assay Kit (Cell Biolabs, Inc., USA). The SCs (1–2 × 10^7^) were rapidly thawed in a bath (37°C), treated with cold 100 mM phosphate buffer (pH 6.0) containing 1 mM EDTA and 0.5% HTAB extraction reagent, and then lysed by sonication. A measure of 1 mM hydrogen peroxide (H_2_O_2_) was added to the SC extract supernatants (obtained by centrifugation at 12,000 rpm for 15 min at 4°C) and incubated for 30 min at room temperature. The MPO activity was defined as the amount of enzyme (one unit) that caused the oxidation of 1 µmole of TNB chromophore per minute at 25°C. Absorbance kinetic was assessed spectrophotometrically at 655 nm (Power Wave XS, BioTek).

### 2.5 Determination of reactive oxygen species/reactive nitrogenous and free radical scavenging activity in milk

The determination of reactive oxygen species/reactive nitrogenous (ROS/RNS) species was measured in milk whey (*n* = 216) following the manufacturer’s suggestion reported in the OxiSelect™ *in vitro* ROS/RNS Assay Kit Green Fluorescence (Cell Biolabs Inc., San Diego, CA, USA). Briefly, the fluorescence intensity is directly proportional to the oxidation of 2′,7′-dichlorodihydrofluorescin (DCFH) activated by ROS/RNS species present in the sample. Data were read against the DCF standard curve (1 concentration range of 0–10,000 nM) using a fluorescence of 480-nm excitation/530-nm emission (CLARIOstar microplate reader, BMG Labtech, Ortenberg, Germany). Data were reported as micromolar of DCF.

Free radical scavenging activity of milk whey (*n* = 216) was determined using 1,1-diphenyl-2-picrylhydrazyl (DPPH) radical scavenging assay as reported in Ciliberti et al. (21), with some modifications. Briefly, DPPH powder was dissolved in methanol to obtain a concentration of 0.1 mM. The DPPH reagent (1,000 μL) was added to the milk whey (200 μL), and the solution was vortexed and incubated at 37°C for 30 min. After the centrifugation step (3,000×*g* for 5 min), the supernatant was added to the plate for reading (520 nm). The absorbance of milk whey was compared to that of the control represented by the DPPH solution (containing methanol). Finally, the formula by Giri et al. (22) was used to obtain the percentage of radical scavenging:


$$\left[\%\mathrm{DPPH radical scavenging activity}\right]=\left[\frac{A-\mathrm{Ax}}{A}\right]*100$$

A = absorbance of the DPPH solution of the control; Ax = absorbance of the milk whey treated with DPPH reagent.

The ROS/RNS quantification was combined with DPPH values in the antioxidant/oxidant balance (AOB) as a biomarker to monitor the antioxidant status of plasma (23).

### 2.6 Statistical analysis

Data were checked for normality using the Kolmogorov–Smirnov test, and the one-way ANOVA of SAS (24) was used to identify the differences based on the three experimental THI classes on the analyzed biomarkers. The significance of the differences was assessed using Tukey’s post-hoc test for multiple comparisons, and the *p*-value of <0.05 was considered statistically significant. A *p*-value of 0.10 < *p* > 0.05 was treated as a tendency.

## 3 Results

### 3.1 Milk cytokine profile

**Figure 2:**
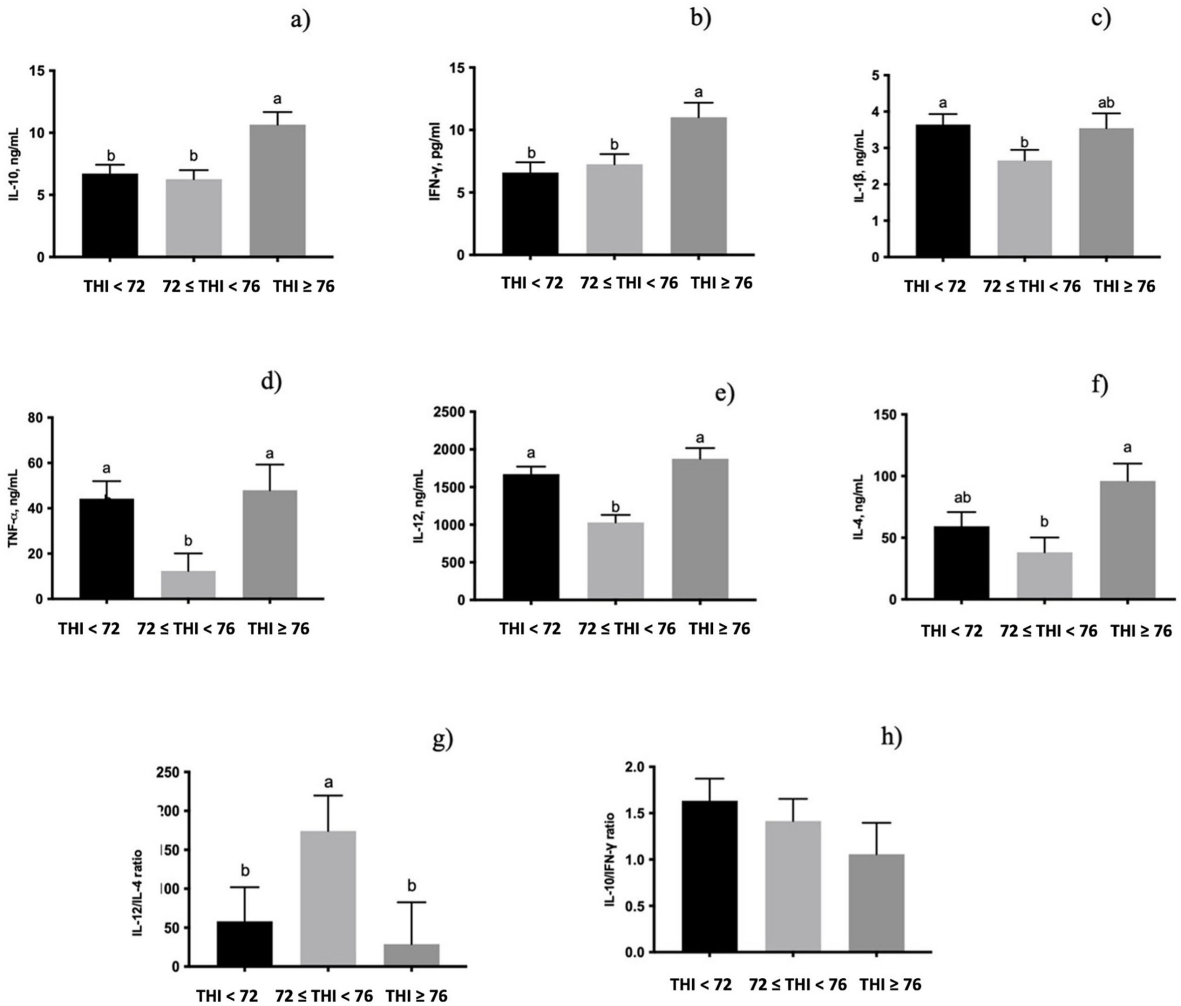
Secretion of (a) IL-10, (b) INF-γ, (c) IL-1β, (d) TNF-α, (e) IL-12, (f) IL-4, (g) IL-12/IL-4 (Th1/Th2 ratio), and (h) IL-10/INF-γ ratio (Th2/Th1 ratio) in buffaloes’ milk whey under three THI classes (THI < 72, 72 ≤ THI < 76, and THI ≥ 76). The data are presented as mean ± SEM. Bars with different letters are significantly different at *p* < 0.05.

All the cytokines measured were significantly influenced by the THI classes (Figure 2). The secretion of both IL-10 (*p* < 0.05, Figure 2a) and IFN-γ (*p* < 0.05, Figure 2b) in milk whey was higher in the class of the THI ≥ 76 than in the other two classes. Moreover, IL-1β (*p* < 0.05, Figure 2c) secretion was lower when the THI increased from ≤72 to <76 range. The level of TNF-α (*p* < 0.05, Figure 2d) and IL-12 (*p* < 0.001, Figure 2e) of milk whey was higher in both the THI < 72 and the THI ≥ 76 classes. On the contrary, the secreted IL-4 (*p* < 0.05, Figure 2f) in milk whey was lower when the THI was in the ≤72 to <76 range than when the THI was ≥76. The IL-4/IL-12 (Figure 2g), measured as a ratio of Th2/Th1, was tendentially influenced by the THI classes (0.10 < *p* > 0.05), showing a higher value in the ≤72 to <76 THI range than in both the THI < 72 and the THI ≥ 76. Conversely, the IFN-γ/IL-10, measured as a ratio of Th1/Th2, was not significantly influenced by the THI classes (Figure 2h).

### 3.2 Milk oxidative status

**Figure 3:**
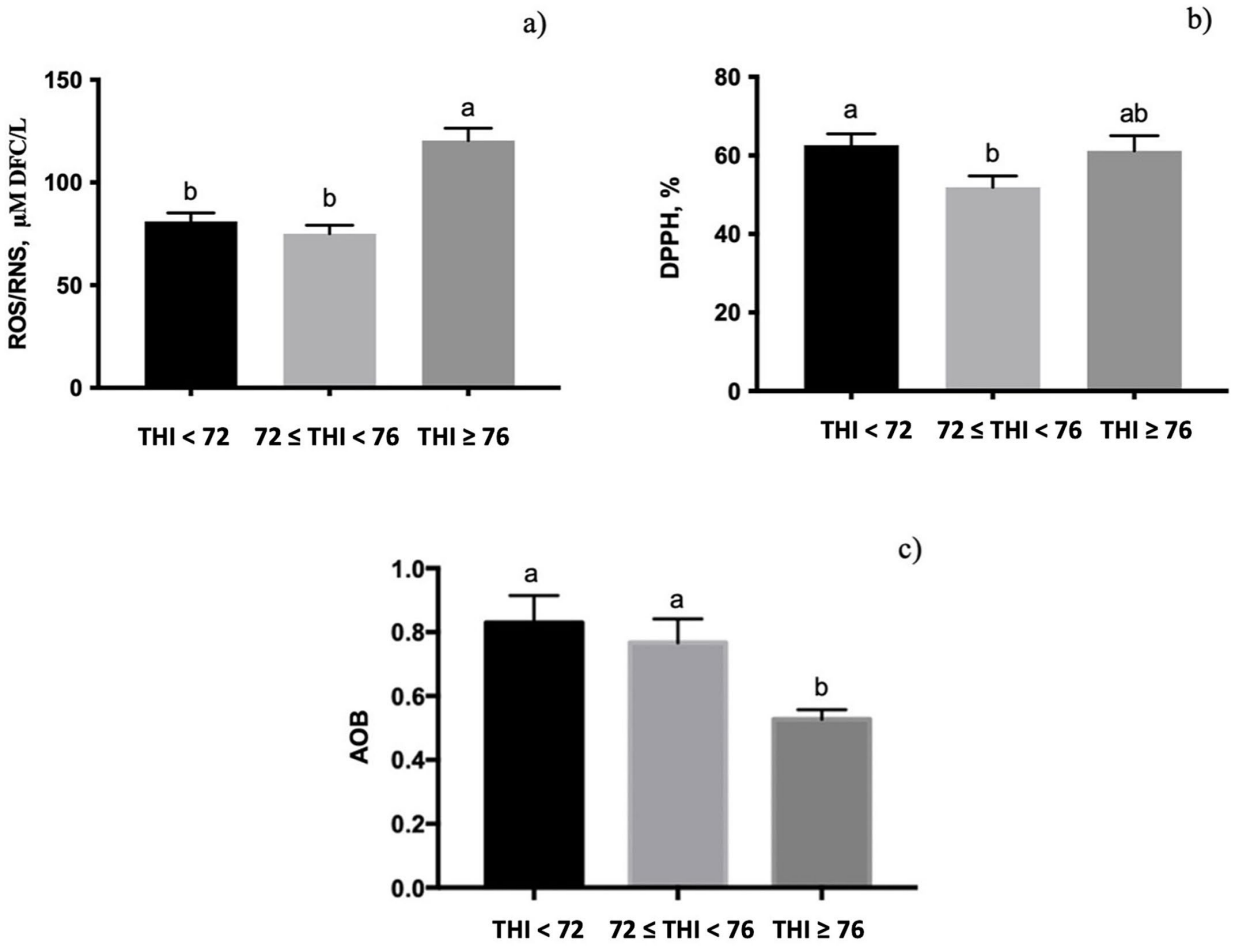
Levels of reactive oxygen and nitrogenous species (ROS/RNS), radical capacity scavenging measured using the DPPH test, and antioxidant–oxidant balance (AOB) measured in buffalo milk whey under three THI classes (THI < 72, 72 ≤ THI < 76, and THI ≥ 76). The data are presented as mean ± SEM. Bars with different letters are significantly different at *p* < 0.05.

The quantification of ROS/RNS in milk whey was significantly affected by the THI class (Figures 3a, *p* < 0.001), displaying the highest value at the THI ≥ 76. The free radical scavenging activity, measured by DPPH, was influenced by the THI; it decreased from the THI < 72 to ≤72 to <76 range (*p* < 0.05, Figure 3b) and returned to the initial levels at the THI ≥ 76. The AOB value showed a significant influence of the THI classes (0.10 < *p* > 0.05, Figure 3c), with lower values at the THI ≥ 76 than at the THI < 72.

### 3.3 Immunophenotyping and myeloperoxidase in somatic cells

Immunophenotyping of milk SCs using DCC showed a significantly higher number of macrophages at the THI ≥ 76 than at the 72 ≤ THI < 76 (Table 1); on the contrary, lymphocytes were fewer at the THI >76 than at the 72 ≤ to <76 range. No significant effects on PMNL numbers were registered among the THI classes. Leukocyte distribution across the THI classes showed that PMNL leukocytes were the most significant SC population, with increasing of THI from the ≤72 to <76 range to the THI ≥ 76 (*p* < 0.05, respectively). A dot plot overlay of the CD11b^+^/CD14^+^ cells subset for each THI class is presented in Supplementary Figure 2.

**Table 1:** Differential somatic cell count (DSCC) of milk buffalo reported in terms of macrophages (CD45^+^, CD14^+^/CD11b^+^ cell subset), PMNLs (CD45^+^, CD11b^+^ cell subset), and lymphocytes (CD45^+^ cells subtracted of macrophages and PMNLs), and surface expression of somatic cells of myeloperoxidase (MPO) (± SE) measured by flow cytometry as affected by three THI classes (THI < 72, 72 ≤ THI < 76, and THI ≥ 76).

Items	THI < 72	72 ≤ THI < 76	THI ≥ 76	*p*-value
Macrophage, % on total CD45^+^	87.33^ab^ ± 11.05	146.00^aB^ ± 38.35	59.89^bB^ ± 8.77	0.047
PMNL, % on total CD45^+^	192.00^b^ ± 80.89	590.14^aA^ ± 174.43	233.00^abA^ ± 70.12	0.068
Lymphocyte, % on total CD45^+^	87.67^ab^ ± 10.99	151.67^aB^ ± 39.12	60.89^bB^ ± 8.95	0.038
MPO, % gated	2.30^a^ ± 0.423	1.43^b^ ± 0.24	1.16^b^ ± 0.09	0.024

^a,b^Different superscript lowercase letters within a column indicate significant differences (*p* < 0.10) among THI classes (*n* = 18 for each milk sampling).

^A,B^Different superscript capital letters within a column indicate significant differences (*p* < 0.05) among the leukocyte population (DSCC) in each THI class (*n* = 18 for each milk sampling).

**Figure 4:**
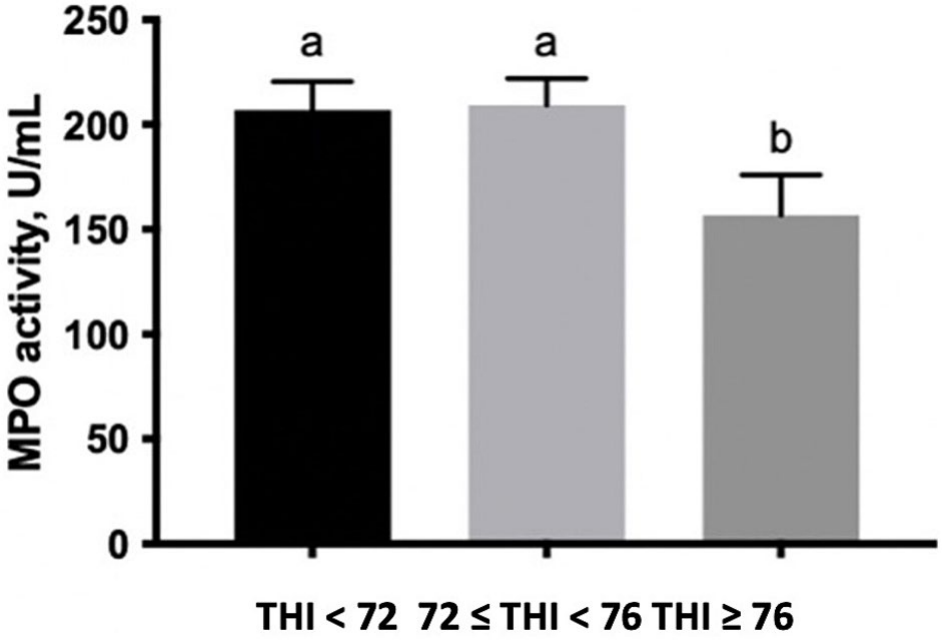
MPO activity (U/mL) measured in buffalo milk somatic cell extracts under three THI classes (THI < 72, 72 ≤ THI < 76, and THI ≥ 76). The data are presented as mean ± SEM. Bars with different letters are significantly different at *p* < 0.05.

The percentage of SC gated, which expresses MPO on the surface, was lower along with the increase of the THI ≥ 72 (Table 1). Finally, the enzymatic activity of MPO measured in the extracts of SC tended to decrease at the THI >76 in comparison to the other two THI classes (0.10 < *p* > 0.05, Figure 4).

## 4 Discussion

The present study is the first report on cytokine profile, oxidative status, and the DCC of buffalo milk as affected by different environmental conditions.

In the mammary gland environment, the immunomodulatory capacity of the cytokine network is very complex. The action of cytokines is considered hormone-like due to their transiently and local production with strong biological activity at extremely low doses; conversely, high levels can be negative to the host (25). In the present study, buffaloes exposed to a THI above 76 exhibited raised levels of IFN-γ in the milk. Interferon-γ is an immunomodulatory cytokine derived from T lymphocytes that enhances macrophage-mediated cytotoxicity against tumor cells and stimulates the synthesis and release of ROS from both macrophages and neutrophils (26). *In vivo* intramammary administration of IFN-γ can enhance the phagocytic and bactericidal capability of neutrophils (27), and the *in vitro* exposure of recombinant bovine IFN-γ can decrease the susceptibility of the mammary gland to infection (28). Previous statements suggest that such a high level of IFN-γ found under the THI ≥ 76 could be associated with a relief of the immune response mediated by IFN-γ in the udder environment.

The IL-10 serves as a bridge between innate and adaptive immune responses by activating the recruitment of monocytes and macrophages through the induction of chemokine-4 and various scavenger receptors, which are responsible for antibody-dependent cell-mediated cytotoxicity, and the phagocytosis of opsonized particles (29, 30). Luttmann et al. (31) suggested that the production of IL-4 could be restricted to the first phases of an initiated Th2 immune response. In the present experiment, the high levels of both the IL-10 and the IL-4 at a THI above 76 suggest a shift toward a Th2-mediated response. However, the concomitant high level of IFN-γ found indicates that the Th1/Th2 shift should be considered balanced, underlining the cross-linking among cytokines and the central role of the cytokine network during the thermotolerance process.

The IL-12, TNF-α, and IL-10 are considered key regulators of the Th1/Th2 balance (32, 33). In the present experiment, both the levels of TNF-α and IL-12 decreased as THI increased from 72 to less than 76. However, this condition was re-established in the highest THI class, probably as an attempt at adaptation to increasing THI. This was further confirmed by the increased tendency of the IL-4/IL-12 ratio (Th2/Th1) in the ≤72 to <76 THI range. In a recent review by Bagath et al. (34), the available results on bovine immune system-related gene expression patterns, which include cytokine patterns during heat stress are not conclusive. Lactating cows exposed to increasing THI showed a reduction of TNF-alpha and IL-10 serum production (35). On the contrary, heat-stressed dairy cows increased the IL-10 expression (36) and its secretion in the plasma (37). Moreover, heat stress reduced the level of TNF-α, IL-1α, and IL-2 cytokines in comparison to the levels registered in cooled dairy cows, coupled with high total immunoglobulin IgM and IgG, thus, demonstrating an alteration of immune responses under heat stress exposition (38). The present data suggest that the cytokines network in milk serves as robust immune biomarkers for defining the immune status of buffalo udders.

Macrophages are the leading cell types found in the milk and lactating mammary glands, which act as active mammary gland phagocytic cells capable of ingesting bacteria and cellular debris (39), and are responsible for the initiation of the immune response (40, 41). Lymphocytes are cells that regulate the induction and suppression of immune responses (25). Milk PMNLs have the main task of defending the udder against invading bacteria at the beginning of an acute inflammatory process (40, 42). In the healthy mammary gland, the SCs are composed predominantly of macrophages and lymphocytes (19, 43, 44). On the contrary, the proportion of PMNLs significantly increases reaching up to 95% of the total leukocyte population during the mastitis infection (42, 45). In addition, it has been revealed the importance of the investigation of the milk cell composition also when the SCC is low (range < 100,000 cells/mL) because elevated proportions of PMNL could indicate inflammatory reactions (19, 43, 44, 46) or at least an active immune response (i.e., triggered by pathogens) (47). In Albenzio et al. (15), no differences in the total SCC were registered in buffalo milk among the different THI classes. On the contrary, the data on DCC showed a decrease in both macrophages and lymphocytes as THI increased from the ≤72 to <76 range to above 76. The SC distribution in each THI class demonstrated the highest proportion of PMNLs in milk from buffaloes exposed to the THI values in the ≤72 to <76 range and above 76. Taken together, all these data suggest that exposure to the THI in the ≤72 to <76 range may trigger an immune response in the mammary glands of buffaloes, leading to changes in the proportion of SCs. The previous statement supports the importance of determining the proportions of individual immune cell populations in milk to describe the actual udder health status of buffaloes.

To add novel information on the immune somatic cell activation in buffaloes, the activity of MPO from SC extracts and the expression of MPO on the SC surface were performed. The MPO plays an important role in innate immunity and has been indicated as a promising marker to evaluate the bovine immune function against bacterial infection (48). Interestingly, a decrease in both the MPO activity in SC extracts and the expression on the cell surface was registered in buffaloes exposed to a THI above 76. Moreover, a reduction in the expression of MPO on the SC surface was detected starting from THI values in the ≤72 to <76 range. Particularly, the reduced activity and the surface expression of MPO along with the increased THI agree with the results on the cytokine network and the balance of Th1/Th2 cytokines, indicating that buffaloes under extreme environmental conditions, as represented by different classes of THI, exhibit proper immune competence. To confirm this assumption, in the previous study by Albenzio et al. (15), no cases of clinical mastitis have been registered among experimental animals.

Oxidative damage is reported to be responsible for immune functional impairment under heat stress conditions (49). Neutrophils and macrophages activated during inflammatory reactions produce the anion superoxide radical (O_2_^−^), which is the major ROS formed mostly within the mitochondria (50). When the production of ROS exceeds the capacity of antioxidant defenses to neutralize them, a condition of oxidative stress occurs (51, 52). It has been demonstrated that buffaloes under heat stress are exposed to oxidative stress due to heat-ROS formation (14). In the present study, exposure of buffalo to a THI above 76 resulted in the highest production of ROS/RNS, coupled with a reduced free radical scavenging capacity at THI values ranging from ≤72 to <76. Accordingly, studies conducted on buffaloes during the summer season showed an increased level of oxidative biomarkers (13) and a decrease in plasma antioxidant enzymes (2), which revealed a reduced total antioxidant capacity to manage the excessive load of ROS produced during the hot season. The AOB index has been previously reported as a reliable biomarker of oxidative stress in both sheep and bovine (18, 21, 23). The AOB measured in the present experiment demonstrated that buffaloes exposed to THI above 76 were under oxidative stress conditions; therefore, it could be supposed an inefficient antioxidant system in milk.

Taking into consideration all the measurements, the exposition to THI classes did not compromise the udder immune competence of dairy buffaloes, showing an immune cell homeostasis condition after a first stage of cellular adaptation to different THI classes. However, an oxidative stress condition emerged that needs to be further elucidated in order to define the physiological response of buffaloes under diverse environmental conditions.

## 5 Conclusion

The present study focused on the evaluation of the cytokine profile, DCC distribution, MPO activity and surface expression, and oxidative status of buffalo milk affected by different environmental conditions. The present data showed that milk cytokine profile was influenced by THI classes, suggesting the key role of those immune biomarkers on buffalo udder health. Moreover, the evaluation of MPO in somatic cells was introduced as a feasible measure of the inflammatory status of the buffalo udder. The oxidative status of milk indicated a condition of oxidative stress when the THI was above 76. This study evidenced an interplay between cytokines profile and immune cell activation confirming that buffaloes are animals with a high capacity to tolerate adverse environmental conditions.

## Data Availability

The raw data supporting the conclusions of this article will be made available by the authors without undue reservation.

## References

[1] Minervino AHH Zava M Vecchio D Borghese A. *Bubalus bubalis*: a short story. Front Vet Sci. (2020) 7:570413. doi: 10.3389/fvets.2020.570413, PMID: 33335917 PMC7736047

[2] Li M Hassan FU Guo Y Tang Z Liang X Xie F. Seasonal dynamics of physiological, oxidative and metabolic responses in non-lactating Nili-Ravi buffaloes under hot and humid climate. Front Vet Sci. (2020) 7:622. doi: 10.3389/fvets.2020.00622, PMID: 33102557 PMC7506138

[3] Mokhber M Shahrbabak MM Sadeghi M Shahrbabak HM Stella A Nicolzzi E. Study of whole genome linkage disequilibrium patterns of Iranian water buffalo breeds using the axiom buffalo genotyping 90K Array. PLoS One. (2019) 14:e0217687. doi: 10.1371/journal.pone.0217687, PMID: 31150486 PMC6544294

[4] Choudhary BB Sirohi S. Sensitivity of buffaloes (*Bubalus bubalis*) to heat stress. J Dairy Res. (2019) 86:399–405. doi: 10.1017/S0022029919000773, PMID: 31787123

[5] Segnalini M Nardone A Bernabucci U Vitali A Ronchi B Lacetera N. Dynamics of the temperature-humidity index in the Mediterranean Basin. Int J Biometeorol. (2011) 55:253–63. doi: 10.1007/s00484-010-0331-3, PMID: 20524014

[6] Segnalini M Bernabucci U Vitali A Nardone A Lacetera N. Temperature humidity index scenarios in the Mediterranean, Basin. Int J Biometeorol. (2013) 57:451–8. doi: 10.1007/s00484-012-0571-5, PMID: 22850789

[7] Kumar VS Kumar RP Harikrishna CH Rani MS. Effect of heat stress on production and reproduction performance of buffaloes-a review. Pharma Innov. (2018) 7:629–33.

[8] Costa A Neglia G Campanile G de Marchi M. Milk somatic cell count and its relationship with milk yield and quality traits in Italian water buffaloes. J Dairy Sci. (2020) 103:5485–94. doi: 10.3168/jds.2019-18009, PMID: 32229124

[9] Upadhyay RC Singh S Kumar A Gupta SK Ashutosh A. Impact of climate change on milk production of Murrah buffaloes. Ital J Anim Sci. (2007) 6:1329–32. doi: 10.4081/ijas.2007.s2.1329

[10] Mishra A Hooda OK Singh G Meur SK. Influence of induced heat stress on HSP70 in buffalo lymphocytes. J Anim Physiol Anim Nutr. (2011) 95:540–4. doi: 10.1111/j.1439-0396.2010.01082.x, PMID: 21091549

[11] Elenkov IJ Chrousos GP. Stress hormones, Th1/Th2 patterns, pro/anti-inflammatory cytokines and susceptibility to disease. TEM. (1999) 10:359–68. doi: 10.1016/S1043-2760(99)00188-5, PMID: 10511695

[12] Scatà MC Alhussien MN Grandoni F Reale A Zampieri M Hussen J. Hyperthermia-induced changes in leukocyte survival and phagocytosis: a comparative study in bovine and buffalo leukocytes. Fron Vet Sci. (2023) 10:1327148. doi: 10.3389/fvets.2023.1327148, PMID: 38322426 PMC10844375

[13] Megahed GA Anwar MM Wasfy SI Hammadeh ME. Influence of heat stress on the cortisol and oxidant-antioxidants balance during oestrous phase in buffalo-cows (*Bubalus bubalis*): thermo-protective role of antioxidant treatment. Reprod Domest Anim. (2008) 43:672–7. doi: 10.1111/j.1439-0531.2007.00968.x, PMID: 18673331

[14] Lan Q Cao Z Yang X Gu Z. Physiological and proteomic responses of dairy buffalo to heat stress induced by different altitudes. Meta. (2022) 12:909. doi: 10.3390/metabo12100909, PMID: 36295811 PMC9609643

[15] Albenzio M Santillo A d'Angelo F di Corcia M Ciliberti MG Marino R. Milk quality of Italian Mediterranean buffalo as affected by temperature-humidity index during late spring and summer. J Dairy Sci. (2024) 107:5343–52. doi: 10.3168/jds.2024-24732, PMID: 38554825

[16] Kelly CF Bond TE. Bioclimatic factors and their measurement. A guide to environmental research on animals. Hoboken, New Jersey, U.S.: National Research Council (1971).

[17] Ciliberti MG Albenzio M Inghese C Santillo A Marino R Sevi A. Peripheral blood mononuclear cell proliferation and cytokine production in sheep as affected by cortisol level and duration of stress. J Dairy Sci. (2017) 100:750–6. doi: 10.3168/jds.2016-11688, PMID: 27865492

[18] Ciliberti MG Albenzio M De Palo P Santillo A Caroprese M. Nexus between immune responses and oxidative stress: the role of dietary hydrolyzed lignin in ex vivo bovine peripheral blood mononuclear cell response. Front Vet Sci. (2020) 7:9. doi: 10.3389/fvets.2020.00009, PMID: 32154273 PMC7045060

[19] Schwarz D Diesterbeck US König S Bruegemann K Schlez K Zschöck M. Flow cytometric differential cell counts in milk for the evaluation of inflammatory reactions in clinically healthy and subclinically infected bovine mammary glands. J Dairy Sci. (2011) 94:5033–44. doi: 10.3168/jds.2011-4348, PMID: 21943754

[20] Cossarizza A Chang HD Radbruch A Abrignani S Addo R Akdis M. Guidelines for the use of flow cytometry and cell sorting in immunological studies. Eur J Immunol. (2021) 51:2708–3145. doi: 10.1002/eji.202170126, PMID: 34910301 PMC11115438

[21] Ciliberti MG Santillo A Caroprese M della Malva A Natalello A Bertino A. Role of hazelnut skin supplementation on plasma antioxidant status and cytokine profile in growing lambs. Front Vet Sci. (2024) 11:1340141. doi: 10.3389/fvets.2024.1340141, PMID: 38362301 PMC10867180

[22] Giri A Kumar Bharti V Kalia S Raj T Chaurasia O. Evaluation of antioxidant status in serum and milk of Jersey cross-bred in different seasons reared under high-altitude stress condition. Biol Rhythm Res. (2019) 50:726–38. doi: 10.1080/09291016.2018.1497769

[23] Ciliberti MG Soccio M Pastore D Albenzio M Sevi A Caroprese M. Antioxidant/oxidant balance: application as a biomarker of the antioxidant status in plasma of ewes fed seaweed Ascophyllum nodosum and flaxseed under high ambient temperature. Small Rumin Res. (2019) 170:102–8. doi: 10.1016/j.smallrumres.2018.11.005

[24] SAS Institute, I. N. C. (2013). SAS (university edition). Midtown Manhattan, New York City.

[25] Sordillo LM Shafer-Weaver K DeRosa D. Immunobiology of the mammary gland. J Dairy Sci. (1997) 80:1851–65. doi: 10.3168/jds.S0022-0302(97)76121-6 9276826

[26] Lawman MJP Campos M Bielefeldt Ohmann H Greibel P Babiuk LA Recombinant cytokines and their potential therapeutic value in veterinary medicine. Comprehensive biotechnology. Babiuk LA Phillips JP Babiuk LA. Phillips JP. London, England: Pergamon Press (1989). 663 p.

[27] Fox LK Liggit HD Yilma T Corbeil LB. The effect of interferon-γ intramammary administration on mammary phagocyte function. J Vet Med Ser B. (1990) 37:28–30. doi: 10.1111/j.1439-0450.1990.tb01022.x, PMID: 2111962

[28] Sordillo LM Babiuk LA. Modulation of bovine mammary neutrophil function during the periparturient period following in vitro exposure to recombinant bovine interferon gamma. Vet Immunol Immunopathol. (1991) 27:393–402. doi: 10.1016/0165-2427(91)90034-A, PMID: 1903898

[29] Di Carlo E Coletti A Modesti A Giovarelli M Forni G Musiani P. Local release of interleukin-10 by transfected mouse adeno-carcinoma cells exhibits pro-and anti-inflammatory activity and results in a delayed tumor rejection. Eur Cytokine Netw. (1998) 9:61–8. PMID: 9613679

[30] Moore KW de Waal MR Coffman RL O'Garra A. Interleukin-10 and the interleukin-10 receptor. Annu Rev Immunol. (2001) 19:683–765. doi: 10.1146/annurev.immunol.19.1.683 11244051

[31] Luttmann W Sengler C Herzog V Balkow S Matthys H Virchow JC Jr. Differential modulation of interleukin-4 and interleukin-13 secretion from human peripheral blood mononuclear cells. Immunol Lett. (1999) 69:225–31. doi: 10.1016/S0165-2478(99)00063-2 10482356

[32] Fearon DT Locksley RM. The instructive role of innate immunity in the acquired immune response. Science. (1996) 272:50–4. doi: 10.1126/science.272.5258.50 8600536

[33] Mosmann TR Sad S. The expanding universe of T-cell subsets: Th1, Th2 and more. Immunol Today. (1996) 17:138–46. doi: 10.1016/0167-5699(96)80606-2, PMID: 8820272

[34] Bagath M Krishnan G Devaraj C Rashamol VP Pragna P Lees AM. The impact of heat stress on the immune system in dairy cattle: a review. Res Vet Sci. (2019) 126:94–102. doi: 10.1016/j.rvsc.2019.08.011, PMID: 31445399

[35] Zhang FJ Weng XG Wang JF Zhou D Zhang W Zhai CC. Effects of temperature–humidity index and chromium supplementation on antioxidant capacity, heat shock protein 72, and cytokine responses of lactating cows. J Anim Sci. (2014) 92:3026–34. doi: 10.2527/jas.2013-6932, PMID: 24879765

[36] Thompson IMT Tao S Monteiro APA Jeong KC Dahl GE. Effect of cooling during the dry period on immune response after *Streptococcus uberis* intramammary infection challenge of dairy cows. J Dairy Sci. (2014) 97:7426–36. doi: 10.3168/jds.2013-7621 25459905

[37] Caroprese M Marzano A Entrican G Wattegedera S Albenzio M Sevi A. Immune response of cows fed polyunsaturated fatty acids under high ambient temperatures. J Dairy Sci. (2009) 92:2796–803. doi: 10.3168/jds.2008-1809, PMID: 19448013

[38] Safa S Kargar S Moghaddam GA Ciliberti MG Caroprese M. Heat stress abatement during the postpartum period: effects on whole lactation milk yield, indicators of metabolic status, inflammatory cytokines, and biomarkers of the oxidative stress. J Anim Sci. (2019) 97:122–32. doi: 10.1093/jas/sky408, PMID: 30346551 PMC6313133

[39] Sordillo LM Nickerson SC. Morphologic changes in the bovine mammary gland during involution and lactogenesis. Am J Vet Res. (1988) 49:1112–20. PMID: 3421535

[40] Oviedo-Boyso J Valdez-Alarcón JJ Cajero-Juárez M Ochoa-Zarzosa A López-Meza JE Bravo-Patiño A. Innate immune response of bovine mammary gland to pathogenic bacteria responsible for mastitis. J Infect. (2007) 54:399–409. doi: 10.1016/j.jinf.2006.06.010, PMID: 16882453

[41] Paape M Mehrzad J Zhao X Detilleux J Burvenich C. Defense of the bovine mammary gland by polymorphonuclear neutrophil leukocytes. J Mammary Gland Biol Neoplasia. (2002) 7:109–21. doi: 10.1023/A:1020343717817 12463734

[42] Paape MJ Wergin WP Guidry AJ Pearson RE. Leukocytes–second line of defense against invading mastitis pathogens. J Dairy Sci. (1979) 62:135–53. doi: 10.3168/jds.S0022-0302(79)83215-4, PMID: 379058

[43] Pilla R Schwarz D König S Piccinini R. Microscopic differential cell counting to identify inflammatory reactions in dairy cow quarter milk samples. J Dairy Sci. (2012) 95:4410–20. doi: 10.3168/jds.2012-5331, PMID: 22818454

[44] Schwarz D Diesterbeck US König S Brügemann K Schlez K Zschöck M. Microscopic differential cell counts in milk for the evaluation of inflammatory reactions in clinically healthy and subclinically infected bovine mammary glands. J Dairy Res. (2011) 78:448–55. doi: 10.1017/S0022029911000574, PMID: 21843398

[45] Kehrli ME Jr Shuster DE. Factors affecting milk somatic cells and their role in health of the bovine mammary gland. J Dairy Sci. (1994) 77:619–27. doi: 10.3168/jds.S0022-0302(94)76992-7, PMID: 8182187

[46] Pilla R Malvisi M Snel GGM Schwarz D König S Czerny CP. Differential cell count as an alternative method to diagnose dairy cow mastitis. J Dairy Sci. (2013) 96:1653–60. doi: 10.3168/jds.2012-6298, PMID: 23332851

[47] Damm M Holm C Blaabjerg M Bro MN Schwarz D. Differential somatic cell count—a novel method for routine mastitis screening in the frame of dairy herd improvement testing programs. J Dairy Sci. (2017) 100:4926–40. doi: 10.3168/jds.2016-12409, PMID: 28365116

[48] Cooray R. Use of bovine myeloperoxidase as an indicator of mastitis in dairy cattle. Vet Microbiol. (1994) 42:317–26. doi: 10.1016/0378-1135(94)90063-9, PMID: 9133057

[49] Sordillo LM Aitken SL. Impact of oxidative stress on the health and immune function of dairy cattle. Vet Immunol Immunopathol. (2009) 128:104–9. doi: 10.1016/j.vetimm.2008.10.305, PMID: 19027173

[50] Valko M Leibfritz D Moncol J Cronin MT Mazur M Telser J. Free radicals and antioxidants in normal physiological functions and human disease. Int J Biochem Cell Biol. (2007) 39:44–84. doi: 10.1016/j.biocel.2006.07.001 16978905

[51] Brenneisen P Steinbrenner H Sies H. Selenium, oxidative stress, and health aspects. Mol Asp Med. (2005) 26:256–67. doi: 10.1016/j.mam.2005.07.004 16105679

[52] Sies H. Biochemistry of oxidative stress. Angew Chem Int Ed Engl. (1986) 25:1058–71. doi: 10.1002/anie.198610581

[53] Caroprese M Ciliberti MG Annicchiarico G Albenzio M Muscio A Sevi A. Hypothalamic-pituitary-adrenal axis activation and immune regulation in heat-stressed sheep after supplementation with polyunsaturated fatty acids. J Dairy Sci. (2014) 97:4247–58. doi: 10.3168/jds.2013-7696, PMID: 24792803

