# Supplementary material for: Cytokine profile, differential somatic cell count, and oxidative status of Italian Mediterranean buffalo milk affected by the temperature–humidity index
Source: Front Vet Sci. 2024 Nov 13;11:1449017. doi: 10.3389/fvets.2024.1449017 (PMC11599857; doi:10.3389/fvets.2024.1449017)
Supplement: Supplementary file 1 [file Data_Sheet_1.docx]

**Cytokine profile, differential somatic cell count, and oxidative status of Italian Mediterranean buffalo milk as affected by Temperature-humidity index**

**Maria Giovanna Ciliberti^1*^, Antonella Santillo^1^, Mariangela Caroprese^1^, and Marzia Albenzio^1^**

^1^Department of Agriculture, Food, Natural Resources, and Engineering (DAFNE), University of Foggia, Via Napoli, 25, 71122 Foggia, Italy

**Supplementary files**

**Table 1.** Min, and max of ambient temperature (°C), humidity (%) and Temperature-Humidity index recorded during the day of each milk sampling and the two days before.

|  | **Temperature, °C** | | **Humidity, %** | | **THI** | |
| --- | --- | --- | --- | --- | --- | --- |
|  | **min** | **max** | **min** | **max** | **min** | **max** |
| -2 day | 12 | 30 | 25 | 88 | 55.42 | 84.15 |
| -1 day | 15 | 30 | 27 | 82 | 58.60 | 83.23 |
| 1^st^sampling | 15 | 29 | 42 | 88 | 58.68 | 82.47 |
| -2 day | 19 | 28 | 42 | 88 | 63.58 | 80.79 |
| -1 day | 16 | 30 | 33 | 94 | 59.77 | 85.08 |
| 2^nd^sampling | 18 | 31 | 33 | 94 | 62.04 | 86.82 |
| -2 day | 21 | 30 | 33 | 73 | 65.45 | 81.84 |
| -1 day | 21 | 32 | 25 | 100 | 64.93 | 89.60 |
| 3^rd^sampling | 18 | 32 | 33 | 69 | 62.04 | 84.21 |
| -2 day | 23 | 34 | 34 | 74 | 67.81 | 88.17 |
| -1 day | 23 | 37 | 25 | 79 | 67.05 | 93.91 |
| 4^th^sampling | 23 | 38 | 22 | 94 | 66.79 | 99.00 |
| -2 day | 21 | 27 | 34 | 73 | 65.52 | 77.24 |
| -1 day | 18 | 29 | 28 | 73 | 61.87 | 80.31 |
| 5^th^sampling | 18 | 29 | 37 | 78 | 62.18 | 81.03 |
| -2 day | 21 | 33 | 32 | 73 | 65.39 | 86.44 |
| -1 day | 17 | 33 | 24 | 83 | 60.68 | 88.28 |
| 6^th^sampling | 21 | 36 | 20 | 78 | 64.61 | 92.11 |

**THI <72 THI >72<76 THI >76**

**
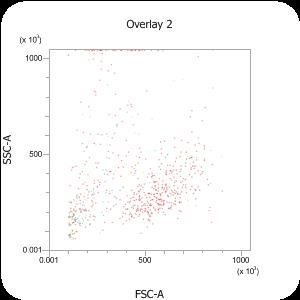
** **
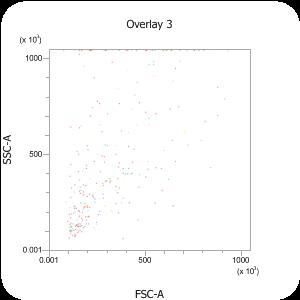
**
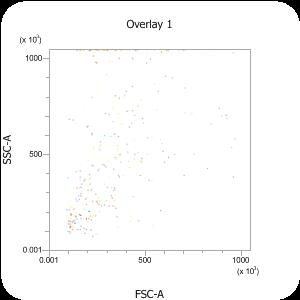


**SSC-A versus FSC-A**

**Figure 1.** Morphological dot plot of positive somatic cell to MPO-FITC antibody.

**THI <72 THI >72<76 THI >76**

**
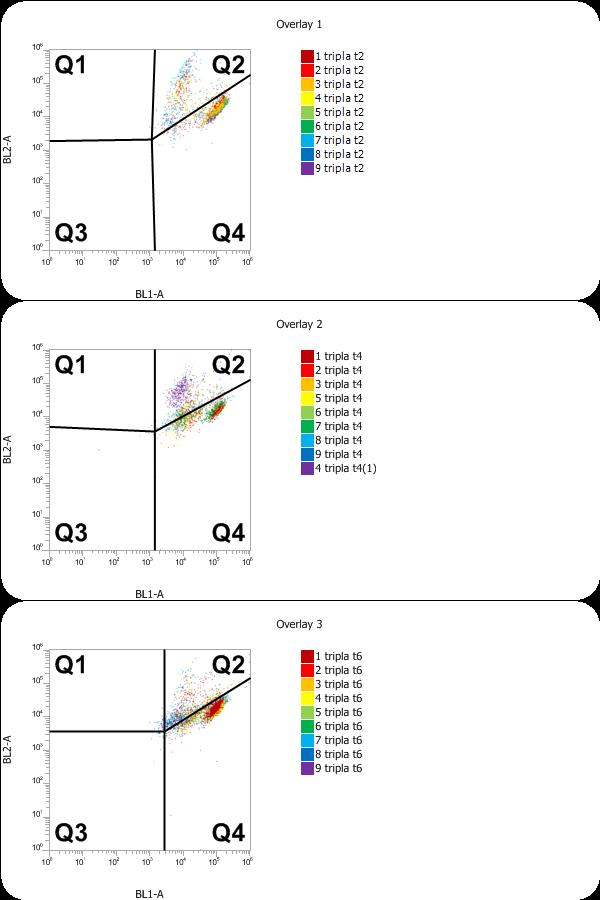
** **
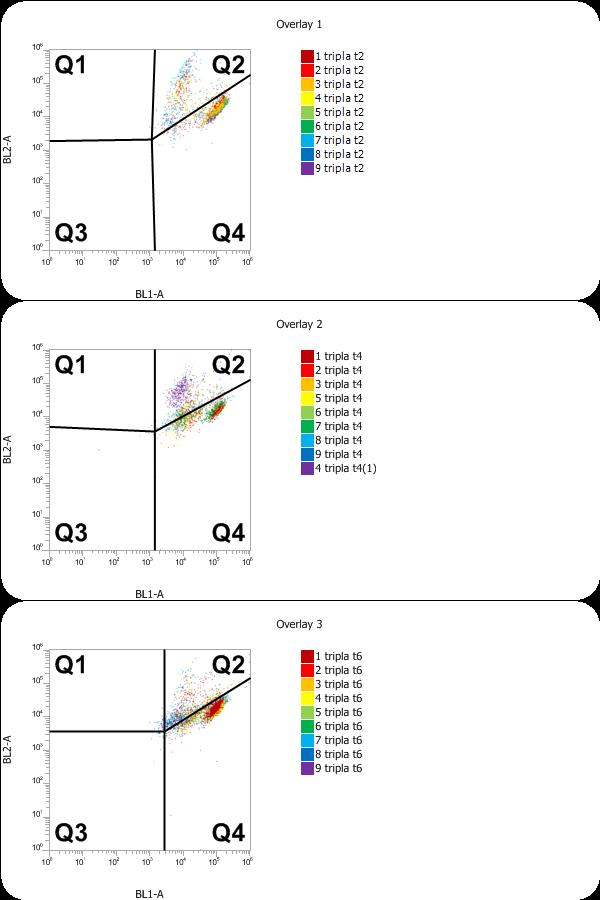

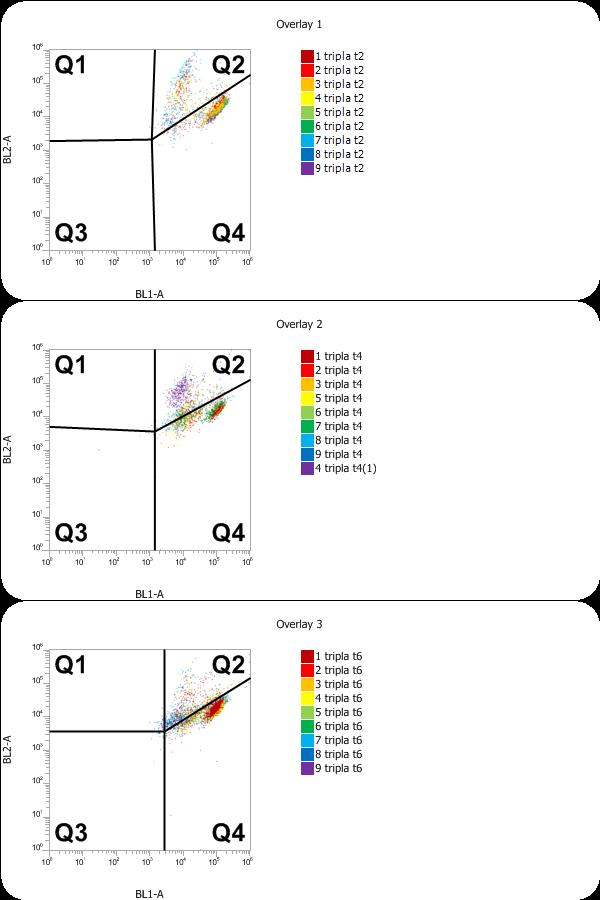
**

**Figure 2.** Overlay of BL1/BL2 (Q2 represents Cd11^+^/Cd14^+^ cell subset of macrophages).
